# Retraction: Soft braces in the treatment of Adolescent Idiopathic Scoliosis (AIS) – Review of the literature and description of a new approach

**DOI:** 10.1186/1748-7161-8-7

**Published:** 2013-05-03

**Authors:** Hans-Rudolf Weiss, Mario Werkmann

**Affiliations:** 1 Orthopedic Rehabilitation Services, Alzeyerstr, 23, Gensingen D-55457, Germany; 2 Orthomed Scolicare, Orthopedic Technical Services, Alzeyerstr, 23, Gensingen D-55457, Germany

## 

The Editor-in-Chief and the Medical Editors at BioMed Central have retracted this article [1] because the first author did not fully declare his association with Koob Scolitech, which holds the rights in and to the Spinealite™ brace and has filed for a patent for that brace. Furthermore, the direct association between Koob Scolitech and the Spinealite™ brace was not made clear in the article. This undeclared competing interest compromised the peer review process.
